# Bilateral Chemical Burns of the Cornea Due to Limewater: A Specific Case

**DOI:** 10.5812/ircmj.2614

**Published:** 2013-01-05

**Authors:** Ebrahim Shirzadeh

**Affiliations:** 1 Department of ophthalmology, Sabzevar University of Medical Sciences, Sabzevar, IR Iran

**Keywords:** Chemical Burns, Bilateral, Cornea

**Figure 1:**
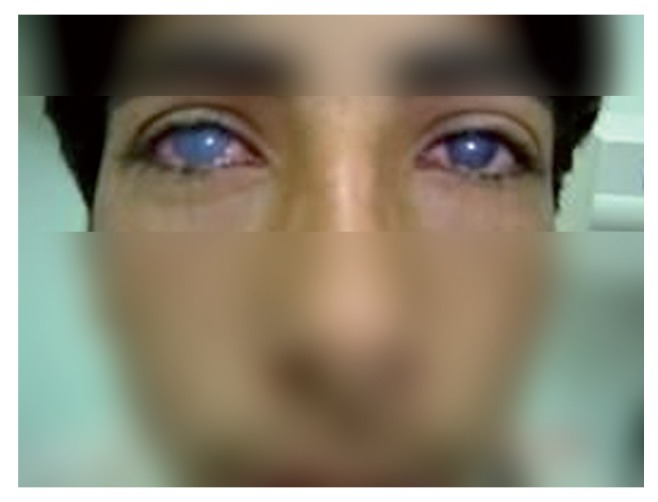
Bilateral Corneal Haze and Red Eye Due to Limewater Burn

Dear Editor,

Eyes Chemical burns may be caused by either acidic or alkaline agents (1). Chemical injury of the eyes may produce extensive damage to the ocular tissues, resulting in permanent unilateral or bilateral visual impairment (2). Damage to the cornea as an impotent clear media and refractive structure require clinicians to rapidly assess the level of injury and to take appropriate steps in order to minimize loss of visual function (3). Although the lime as a common alkaline agent has harmful effects to the eye tissues and chemical burns of eye tissues due to lime have been reported (4) but we here present a specific case of bilateral chemical injury of the cornea. An 18-year-old Iranian adult male was admitted at hospital for assessment of chemical burns of both eyes. Past medical history and familial history were normal. The patient was a shepherd and upon returning home from field, he encountered a few children in the alley playing together. A plastic bottle (1.5 liter) with a milky solution has been offered to him as a hoax. The bottle was similar to buttermilk but it contained limewater. The bottle explosion happened as early as it was picked up from ground, and chemical burn of both eyes followed. The patient was dispatched to the hospital for treatment. On physical examination, findings were as follow: Visual acuity (dist) in the right eye was 1m counting finger (CF) and in the left eye was 1.5m CF. Eye movements in both eyes were full. On Slit Lamp Exam: Conjunctiva/Sclera was injected; bilateral corneal edema and haze without any epithelial defect and limbal ischemia was obvious (Figure 1). Intraocular pressure (IOP) in both eyes was 13 mm Hg. Anterior chambers were deep and iris details were visible. No other ophthalmic lesions were seen. After 2.5 weeks treatment and follow up, his visual acuity was 1m CF in the right eye, and nearly 2m CF in the left eye. The corneal opacity did not improve significantly but conjunctive injection reduced. Patient follow-up for more than four years revealed that corneal haze in the left eye relatively improved but the right eye needed corneal transplantation. Alkaline agents like limewater are particularly damaging as they have both hydrophilic and lipophilic properties, which allow them to rapidly penetrate cell membranes and enter the anterior chamber (1). Effective emergency measures must be instituted immediately followed by careful clinical evaluation in order to recognize and treat problems as they arise (5). The first step in the treatment of chemical injuries to the eyes is immediate, thorough, and if necessary, prolonged irrigation (6). The speed, at which initial irrigation of the eye begins, has the greatest influence on the prognosis and outcome of eye burns. Often tap water is the only irrigation solution available. It produces quite good results if used immediately. Thus, water is commonly recommended as an irrigation fluid as to use by this case. However, water is hypotonic to the corneal stroma. The osmolarity gradient causes an increased water influx into the cornea and the invasion of the corrosive substance into deeper corneal structures. Therefore, purpose-designed rinsing solutions are preferable if available (7, 8). Inhibition of inflammatory mediators by topical corticosteroid therapy is the most effective therapy for acute inflammation. Thus, topical corticosteroids reduce the inflammatory destruction of the surviving structures caused by chemical agents and promote faster epithelial healing despite their concomitant inhibition of extracellular matrix production. So we too prescribed Dexamethasone 1% eye drops four times a day for two weeks. Bacterial infections due to chemical burns of eyes are rare but can cause severe complications and should be prevented. Thus, prophylactic topical antibiotic therapy is recommended and in this case ciprofloxacin 1% eye drops four times a day prescribed. Dry eyes and rough surfaces can be major problems in late stages of the chemical eye injury, and ongoing mechanical damage should be prevented by keeping the surfaces smooth and moist (8). For these purpose preservative free artificial eye drops of artelic was the moisture medication in this case. Also tropicamid 1% eye drops three times a day was used as cycloplagic agent to prevent ciliary spasm. Since bilateral corneal edema and haze without any epithelial defect, limbal ischemia, and visible iris details was nearly good prognosis sign thus, further treatment was not required. In this case more than four years close follow-up showed nearly total improvement of haze in the left cornea and happening of surface irregularity, opacity and vascularization in the right cornea. Therefore this signified more severe damage to the right eye and longtime follow-up is essential for detecting late onset complication, prognosis of damage to different tissues and choice of final treatment. Severe damage to the right eye may be due to right handedness of patient and it’s probably closeness of bottle of limewater to his right eye and/or face turning. Unfortunately long time follow-up revealed that the right eye needs unwanted corneal transplantation but this procedure too has poor prognosis due to limbal stem cell deficiency and conjunction of cornea. In conclusion, limewater in plastic bottles may mimic the buttermilk and explosion of bottles containing limewater can cause unwanted chemical injuries in both eyes and visual impairment. Thus, children hoaxing with limewater are very hazardous for eyes.
